# Label-Free Detection of Cancer Biomarkers Using an In-Line Taper Fiber-Optic Interferometer and a Fiber Bragg Grating

**DOI:** 10.3390/s17112559

**Published:** 2017-11-06

**Authors:** Dandan Sun, Yang Ran, Guanjun Wang

**Affiliations:** 1 College of Physics and Electronic Engineering, Shanxi University, Taiyuan 030006, China; 2 Guangdong Provincial Key Laboratory of Optical Fiber Sensing and Communications, Institute of Photonics Technology, Jinan University, Guangzhou 510632, China; tranyang@jnu.edu.cn; 3 School of Information and Communication Engineering, North University of China, Taiyuan 030051, China; wangguanjun@nuc.edu.cn

**Keywords:** fiber taper interferometer, FBG, optical biosensor, cancer biomarker

## Abstract

A compact and label-free optical fiber sensor based on a taper interferometer cascaded with a fiber Bragg grating (FBG) is proposed and experimentally demonstrated for detection of a breast cancer biomarker (HER2). The tapered fiber-optic interferometer is extremely sensitive to the ambient refractive index (RI). In addition, being insensitive to the RI variation, the FBG can be applied as a temperature thermometer due to its independent response to the temperature. Surface functionalization to the sensor is carried out to achieve specific targeting of the unlabeled biomarkers. The result shows that the proposed sensor presents a low limit-of-detection (LOD) of 2 ng/mL, enabling its potentials of application in early diagnosis on the breast cancer.

## 1. Introduction

Breast cancer has become one of the most common malignancies, which is a serious threat to women’s health with an incidence rate of 7–10% of systemic cancer. Therefore, early diagnosis on the suspect patients is of utmost importance. Cancer biomarkers are typically found in body fluids such as blood, serum, urine, and tumor cells [1,2]. Measurement on the content of certain markers can contribute significantly to the determination of the presence or progression of certain types of cancer [3]. HER2 is a well-documented biomarker that is related to tumor cell proliferation with overexpression in 20–30% of human breast cancers [4]. Fluorescent labels or chromosomes [5,6,7] are utilized in some effective and available methods of breast cancer biomarker detection that can provide exceptional sensitivity down to the single molecule level. However, challenges still exist in terms of cost, complexity, and labeling heterogeneity. Therefore, demands for new tools are increasing to facilitate and accelerate unlabeled approaches for detecting cancer biomarkers.

New types of optical fiber biosensors have been developed for detecting biomarker in a rapid and label-free manner, showing great potential because of their compact size, easy implementation and diversity in structure. As a promising fiber device, providing high sensitivity due to strong evanescent fields, microfiber interferometer biosensors have been effectively functionalized with biorecognition molecules (oligonucleotides or antibodies) and employed to recognize specific target analytes [8,9]. However, in such microfiber interferometer biosensors, temperature cross-sensitivity usually exists, which leads to undesired perturbation during the process of biological sensing measurement [10]. Therefore, it is essential to detect the target concentration and temperature simultaneously, especially in a real-time monitoring system.

Here we propose a compact optical fiber based on a taper interferometer embedded in a fiber Bragg grating (FBG) for HER2 biomarker detection. The FBG, which is insensitive to RI variation, can be applied as a temperature thermometer due to its independent response to the temperature. The tapered fiber-optic interferometer sensor can be easily fabricated by drawing a commercial single-mode fiber (SMF) down to 15 µm in diameter and is extremely sensitive to ambient refractive index (RI) with a sensitivity of 2333 nm/RIU. Surface functionalization is accomplished for achieving target specific detection. The results show that the proposed sensor presents the lowest detectable concentration of 2 ng/mL. The present work provides great potential for applications in early diagnosis of breast cancer.

## 2. In-Line Taper Fiber-Optic Interferometer and FBG

### 2.1. Principle and Analysis

The Bragg phase matching condition [11] is

(1) $$\lambda_{B}=2n_{eff}\Lambda$$

where $\lambda_{B}$, $n_{eff}$, and Λ are the resonant wavelength, the effective refractive index, and the grating pitch, respectively. For working on fundamental core modes, the FBG inscribed in standard single mode fiber (SMF) is inherently insensitive to ambient RI. FBG is commonly used as a temperature sensor. The temperature response of FBG can be expressed [11] by

(2) $$\frac{d\lambda_{B}}{dT}=\lambda_{B}\left(\alpha +\beta \right)$$

where $\alpha =\frac{1}{\Lambda }\cdot \frac{d\Lambda }{dT}$ is the thermal expansion coefficient, ~0.55 × 10^−6^/°C; $\beta =\frac{1}{n_{eff}}\cdot \frac{dn_{eff}}{dT}$ is the thermo-optic coefficient, ~6 × 10^−6^/°C of the SMF. Hence, the resonant wavelength of the FBG changes with the temperature.

The tapered fiber-optic interferometer sensor comprises the central uniform waist region and two transition regions on both sides. The fundamental mode and high order modes are excited by one transition region, then after propagating along the central uniform waist region, two modes are coupled at the other transition region. The interferometric fringes can be considered a result of the phase difference between two modes over the taper. Under the condition that the fiber can keep cylindrical symmetry, the couplings of different HE_1n_ local modes are mainly in the transition regions. We find that the coupling strength of the fundamental HE_11_ mode to HE_12_ mode is much stronger than other higher order modes [12]. The output intensity of tapered fiber-optic interferometer sensor can be described [13] by

(3) $$I=I_{co}+I_{cl}+2\sqrt{I_{co}I_{cl}}\cos\left(\Delta \phi \right)$$

where $I_{co}$ and $I_{cl}$ are the intensities of the HE_11_ and HE_12_ modes, respectively. When forming interferometric fringes, the phase difference satisfies Δϕ=2(k+1)π, where k is the interference order.

Normally, the external RI sensitivity can be expressed [14] by

(4) $$\frac{d\lambda }{dn_{ex}}=\frac{\lambda }{\Gamma }\left(\frac{1}{\Delta n}\frac{d\Delta n}{dn_{ex}}\right)$$

where $\Gamma =1-\frac{\lambda }{\Delta n}\frac{d\Delta n}{d\lambda }$ is the dispersion factor, which is typically a negative value for silica optical microfiber, and characterizes the relationship between the index difference and the wavelength. Δn is the mode index difference between HE_11_ and HE_12_ modes. The term in the bracket shows the dependence of the index difference on the external RI change. When the external RI increases, the higher-order mode has a larger variation rate because of the larger energy fraction in the evanescent field. Thus, the index difference decreases with external RI increases, and subsequently the term in the bracket is also negative. As a result, the transmission wavelength red-shifts with increasing RI.

Similarly, the temperature response can be obtained [14] as

(5) $$\frac{d\lambda }{dT}=\frac{\lambda }{\Gamma }\left(\alpha +\frac{\beta_{si}}{\Delta n}\frac{d\Delta n}{dn_{si}}\right)$$

where α is the thermal-expansion coefficient and $\beta_{si}=dn_{si}/dT$ is the thermo-optic coefficient as discussed above. Through calculating the alteration of index-difference as $n_{si}$ change, we obtain that the index difference Δn decreases with the increase of $n_{si}$. Thus, the second item in the bracket is around −1.04 × 10^−4^/°C. Due to the dispersion factor is also negative [12,14], the transmission dip red-shifts with the rise of temperature. Using numerical mode simulation (COMSOL), the numerical values of Δn and dΔn/dλ are obtained, and the Γ of 1520 nm is thus −0.91. The calculated temperature sensitivity around 1520 nm is about 17.3 pm/°C, which is close to the measured result as shown in Section 2.2.

### 2.2. Sensor Fabrication and Characterization

Here in this work, the method of Bragg gratings written in single-mode fiber (SMF) is the phase-mask technique by a 193 nm ArF excimer laser (0.6 W, 200 Hz). The period of phase mask plate is 1070.49 nm. The length of inscribed grating is ~3 mm.

The tapered fiber-optic interferometer is fabricated by drawing SMF near the FBG down to the micrometer range with a commercial fusion splicer (Fujikura, Japan, FSM100P+) [15]. The fixed electrodes used as a high-temperature heat source generates the continuous arc discharge to heat the fiber over a localized region. The desired geometry is formed by controlling the speed of right and left motors with combination of arc intensity. In this work, the length and diameter of the microfiber are about 3 mm and 15 µm, respectively. It is straightforward to implement good reproducibility with the proposed method. 

**Figure 1:**
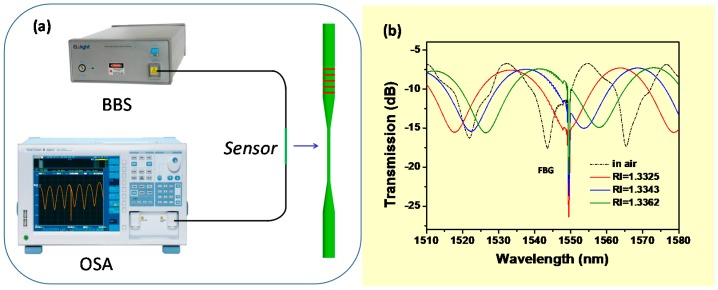
(**a**) The experimental setup including this sensor structure of the tapered fiber-optic interferometer with a fiber Bragg grating (FBG) and (**b**) the transmission spectrum for different RI values.

Figure 1a illustrates the experimental setup, including this sensor structure of the tapered fiber-optic interferometer with FBG in the enlarged portion. The broadband source (BBS, 1250–1650 nm) emits light through this sensor to an optical spectrum analyzer (OSA) with a resolution of 0.01 nm, which records transmission spectra. As a previous paper [16] shows, sensor probes are fixed in PDMS (polydimethylsiloxane)-based micro-fluidic channels (width 200 μm by height 150 μm) designed specifically for biosensing tests.

**Figure 2:**
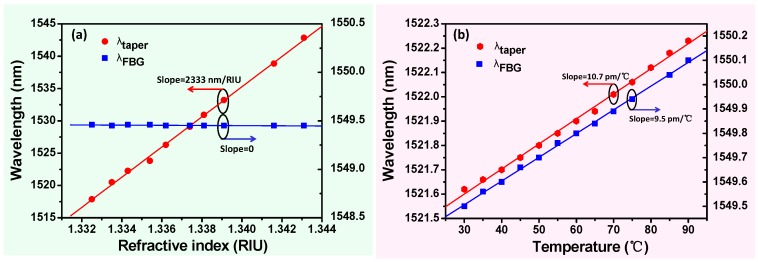
Wavelength response of the tapered fiber-optic interferometer and FBG versus (**a**) RI and (**b**) temperature.

Figure 1b displays the transmission spectrum of the tapered fiber-optic interferometer integrated with the FBG for different RI values, i.e., 1 (air), 1.3325, 1.3343, and 1.3362. The refractive index characterization is performed by immersing the sensor into the sucrose solution. The transmission spectrum appears “red-shifted” due to increasing RI, but the resonance wavelength of FBG is essentially unchanged (Figure 2a). The results show the wavelength response of this sensor surrounding RI (1.332–1.344), which obtains an RI sensitivity of 2333 nm/RIU for the tapered fiber-optic interferometer and an RI sensitivity of about zero for the FBG. Figure 2b illustrates the temperature change (30–90 °C) of the sensor, which increases linearly with temperature in the wavelength response of the tapered fiber-optic interferometer and the FBG. The temperature sensitivity of the tapered interferometer ($K_{taper}$) is 10.7 pm/°C, and the temperature sensitivity of the FBG ($K_{FBG}$) is 9.5 pm/°C. It should be noted that the response to the temperature of FBG is independent relative to that of the taper interferometer. Thus, an FBG component can be regarded as a temperature thermometer for monitoring temperature perturbations during the biological sensing process. In the meantime, the wavelength shift information of transmission spectrum interferometric fringe is applied to monitor the biomolecular interactions. 

Temperature perturbation can be obtained by the following equation:

(6) $$\lambda_{taper}(T)=\left(K_{taper}/K_{FBG}\right)*\lambda_{FBG}\left(T\right)$$

where $\lambda_{taper}(T)$ is the temperature-induced tapered interferometer wavelength shift, while $\lambda_{FBG}\left(T\right)$ is the FBG temperature-induced wavelength shift.

## 3. Results and Discussion

**Figure 3:**
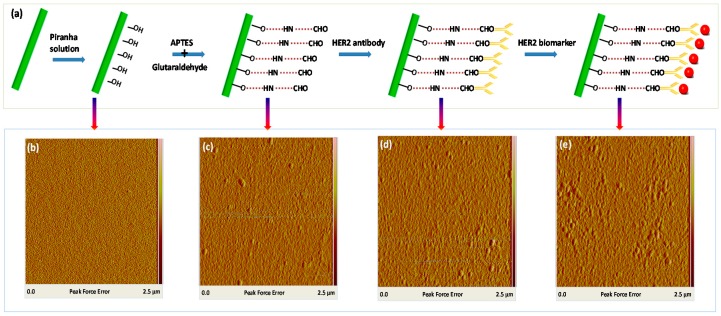
(**a**) The scheme of surface functionalization of the tapered fiber-optic interferometer with FBG biosensors; (**b**–**e**) AFM images of the fiber surface in the conjugation process: (**b**) bare fiber; (**c**) silanization and crosslinking reaction (APTES + glutaraldehyde); (**d**) HER2 antibody; (**e**) biomarker detection).

The covalent immobilization method [17,18] is used as described in Figure 3a, which can be divided into the following five steps:

(1) Treatment with a freshly prepared piranha solution (H_2_SO4/H_2_O_2_ = 70:30, v/v), which is processed for 30 min, rinsed with de-ionized (DI) water, and dried with nitrogen
(2) Reaction of the fiber surface 5% 3-aminopropyl-triethoxysilane (APTES) in ethyl alcohol, which is processed for 15 min, thoroughly rinsed with DI water, and dried.
(3) Crosslinking reaction of 2.5% glutaraldehyde solution for 30 min, which is thoroughly rinsed with DI water and dried.
(4) Immobilization of the HER2 antibody with a concentration of 10 µg/mL, which is processed for 1 h and thoroughly rinsed with DI water.
(5) Immersion in 1% bovine serum albumin (BSA) solution in phosphate buffer saline (PBS) buffer for 5 min to block the non-reacted sites to minimize nonspecific adsorption.

The piranha solution produced reactive hydroxyl groups on the fiber surface. The fresh 3-aminopropyl-triethoxysilane (APTES) in ethyl alcohol produced a free amine group (NH2^−^) and the fiber surface was effectively immobilized. The silanized fiber became available for further reaction with aldehyde groups (-CHO) of glutaraldehyde to form imines. Then the fiber was washed with DI water for 2 min, which worked to remove the excess adsorbed components, and dried with nitrogen. The fiber was dipped in HER2 antibody solution for 60 min and washed in DI water. Next, the fiber, with this bio-recognition layer, eliminated the non-reacted sites, minimizing the nonspecific adsorption in the BSA solution. Finally, the functionalized biosensor probe was ready for HER2 biomarker detection. Figure 3a presents the conjugation process, which corresponds to the fiber surface morphology using atomic force microscopy (AFM) in Figure 3b–e. The Image Ra values (arithmetic average of the absolute values of the surface height deviations measured from the mean plane) of roughness of AFM images are 3.44 nm, 3.48 nm, 3.57 nm, and 3.83 nm, respectively, in Figure 3b–e. The smooth surface (Figure 3b) was slightly roughened after the APTES and glutaraldehyde modification (Figure 3c). After conjugation with HER2 antibody, the fiber surface was further roughened (Figure 3d). Finally, after HER2 biomarker detection, the fiber surface was roughest with agglomerative bio-particles.

**Figure 4:**
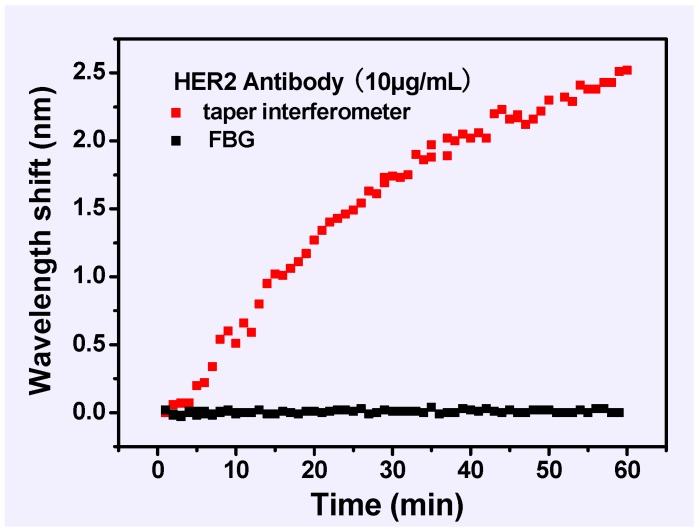
The real-time wavelength response of surface-functionalized biosensors in the HER2 antibody solution (10 µg/mL).

Figure 4 presents the wavelength responses of surface-functionalized biosensors over the above fourth step of the testing procedure (immobilization of the HER2 antibody). The wavelength response was observed in real time by making data recordings every minute 60 s intervals. Due to antibody binding over time, which induced an increase in the RI of the fiber surface, the wavelength of the tapered fiber-optic interferometer drifted by ~2.5 nm. 

**Figure 5:**
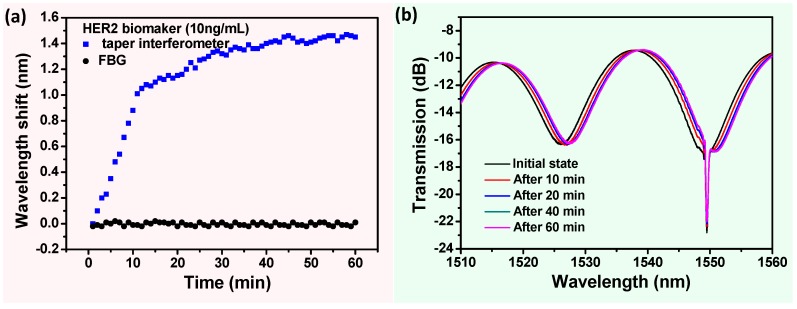
(**a**) The real-time wavelength response and (**b**) the measured transmission spectra of surface-functionalized biosensors in the HER2 biomarker solution (10 ng/mL).

Similarly, in the process of HER2 biomarker detection with a concentration of 10 ng/mL, the wavelength shifts, and spectral responses are demonstrated in Figure 5. In detail, the wavelength response was recorded in real time for 60 min. The spectral response in the biomarker solution (Figure 5b) was monitored between the initial state and the ultimate state (after 60 min) at different times. The wavelength dips of the tapered fiber-optic interferometer in the initial state were taken as the baseline for each measurement. Regarded as a temperature thermometer, the FBG was insensitive to RI variation, while the wavelength-shift-induced temperature ($\lambda_{FBG}\left(T\right)$) was almost zero (Figure 4 and Figure 5). These results show that the proposed sensor is free from temperature perturbations throughout the biological sensing process, which is in good agreement with Equation (6) in Section 2.2. Figure 5a shows a rapid red-shift in wavelength shift of 1.02 nm over the first 10 min of immersion, which then gradually become stabilized, and the whole process drifts by ~1.45 nm. The above results indicate the presence of the HER2 biomarker in the vicinity of the sensing region, which is related to the RI change of the surface biosensor due to the high affinitive and specific reactions between the HER2 antibody and biomarker.

**Figure 6:**
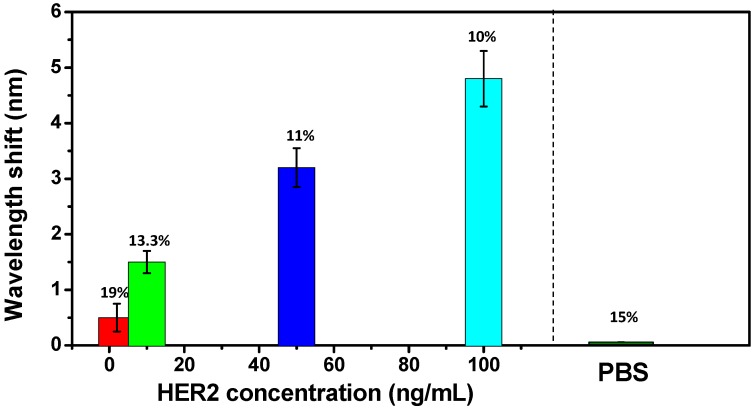
Statistical sensing results over repeat measurement with different concentrations of HER2 biomarker and PBS buffer solution in the absence of HER2 biomarker.

Finally, it should be noted that, the used probe was developed to be disposable, because sensor fabrication is reproducible with the proposed method as shown in Section 2.2. The deposition steps were performed again following the immobilization process of Figure 3. The response of biomarker detection was re-recorded. Figure 6 shows the wavelength shifts of the repeated experimental results with different concentrations of HER2 biomarker (2 ng/mL, 10 ng/mL, 50 ng/mL, and 100 ng/mL) and with PBS buffer solution without HER2 biomarker after 60 min. Statistical sensing results present the average outputs and errors (relative standard deviation (RSD)). 

## 4. Conclusions

A compact and label-free fiber-optic biosensor based on taper interferometer embedded in an FBG has been demonstrated for cancer biomarker detection. Being insensitive to RI variation, an FBG component can be regarded as a temperature thermometer for monitoring temperature perturbations during the biological sensing process. Meanwhile, the taper interferometer is extremely sensitive to external RI and the wavelength shift information is applied to monitor the biomolecular interactions. With the help of surface functionalization, the target cancer biomarker can be specifically detected with the lowest detectable concentration of 2 ng/mL. The proposed biosensor opens a good platform for highly sensitive and specific measurement in early diagnosis of breast cancer.
